# Total flavones from *Sonchus arvensis L.* ameliorate colitis by adjusting the gut microbiota

**DOI:** 10.1080/07853890.2023.2292246

**Published:** 2023-12-13

**Authors:** Yachao Ren, Shenghua Hou, Jing He, Naidan Chang, Zecai Zhang, Yulong Zhou

**Affiliations:** a School of Chemistry and Chemical Engineering, Tianjin University of Technology, Tianjin, China; b Harbin Medical University-Daqing, Daqing, China; c College of Animal Science and Technology, Heilongjiang Bayi Agricultural University, Daqing, China

**Keywords:** *Sonchus arvensis* L., ulcerative colitis, gut microbiota, dextran sulphate sodium, flavones

## Abstract

**Objective:**

*Sonchus arvensis L.* is traditional Chinese food and medicine. We investigated protective effects of flavones from* Sonchus arvensis L.* (SAF) on colitis induced by dextran sulfate sodium (DSS) in mice by regulating gut microbiota (GM).

**Method:**

C57BL/6 mice were divided randomly: control group (CL); DSS group (ML); positive control + DSS group (AN); SAF + DSS (FE) group. The protective effects of SAF on ulcerative colitis (UC) were estimated by food intake, water intake, bodyweight loss, diarrhea, blood in stools, colon length, histology, disease activity index (DAI) score, and blood parameters. The sequencing of 16S rRNA gene was detected to investigate effect of SAF on GM.

**Results:**

SAF attenuate bodyweight loss significantly. The DAI score was lower in FE group than that in ML group. Colon length was improved significantly in ML group. Pathologic changes could be ameliorated after SAF was administered to UC mice. SAF improved blood parameters of model mice. 16S rRNA sequencing revealed that it was very important to ameliorate colitis with bacteria of the phylum Verrucomicrobiota, class Verrucomicrobiae, order Verrucomicrobiales, family Akkermansiaceae, and genus *Akkermansia*.

**Conclusion:**

The SAF protective effect against colitis induced by DSS in mice may have a connection with GM diversity.

## Introduction

Inflammatory bowel disease (IBD) is a chronic, recurrent disease of the digestive system [1,2]. The World Health Organization has stated that IBD is an intractable disease [3]. IBD includes ulcerative colitis (UC) and Crohn’s disease, which are similar in that they cause inflammation and digestive disorders. The typical symptoms of UC are abdominal pain, diarrhoea and stool bleeding [4]. UC increases the risk of patients eventually developing colon cancer [5,6]. So, UC seriously affects the study, life and work of patients [7].

The primary pathologic mechanism of UC is not known. The aetiology of UC is related to environment, lifestyle, immune function, genes and the gut microbiota (GM). Many reports have indicated that GM dysbiosis is related to IBD [4]. The diversity and stability of communities in the GM are lower in patients with a chronic inflammatory disease [8]. Aminosalicylates, corticosteroids, prebiotics, antibiotics, as well as immunosuppressive drugs have been used to treat UC. Among them, the use of antibiotics has had a relatively good therapeutic effect [9–11]. However, long-term antibiotic treatment can result in the resistance of intestinal microorganisms to the effects of antibiotics. Transplantation of faecal microbiota is a new and safe method to treat UC, but it can lead to the spread of unknown pathogens [12]. Therefore, a new type of treatment is needed urgently.

It has been reported that traditional Chinese medicine can be used to treat diseases by regulating the homeostasis of human intestinal flora. *Sonchus arvensis* L. is Asteraceae family, which is a food and medicine named ‘Ju Mai Cai’ in Chinese [13]. *S. arvensis* L. is distributed mainly in the north of China [13]. Previously, we demonstrated that *S. arvensis* L. extract had a protective effect against dextran sulphate sodium (DSS)-induced UC in mice, and that these protective effects may be connected with GM diversity. The major constituents of *S. arvensis* L. extract are flavones [14]. Flavones (and their derivatives) have been reported to have anti-oxidation, anti-aging, anti-inflammatory, antiviral, anti-tumour and immunoregulatory activities, as well as to regulate the composition of gut microbes [15–22]. We hypothesized that flavones may be the main components of *S. arvensis* L. extract that inhibit colitis and which shape GM composition. Therefore, we designed a study to investigate if the flavones in *S. arvensis* L. extract (SAF) could ameliorate colitis and postulated the underlying molecular mechanism of action.

## Materials and methods

### Materials

*S. arvensis* L. were acquired from local market. The morphological characteristics and was observed to authenticate by comparing them with the pattern specimen. Our research team has already preserved the voucher specimens. DSS was supplied by MP Biomedicals (Irvine, CA, USA). Rutin was obtained from the National Institutes for Food and Drug Control (Beijing, China). All other chemicals were provided from MilliporeSigma (Burlington, MA, USA).

### Animals

The study protocol was approved by the ethics committee of Harbin Medical University and ethical number is DWKJXY2023015. Male C57BL/6 mice (21-23 g) were obtained from Charles River Laboratories (Beijing, China), which were housed in a specific pathogen-free environment.

### Preparation of SAF

The whole plant of *S. arvensis* L. was cut into pieces. Pieces (100 g) were macerated in 50% ethanol solution followed by three-time ultrasound agitation for 30-min each. The extraction was centrifugated to collect the liquid supernatant, and the liquid supernatant was extracted using petroleum ether. Then, the ethanol solution was collected and concentrated by a rotary evaporator. Next, the concentrated extract was dried further in a freeze-dryer (100FG/A; Scientz, Ningbio, China) and then stored at −20 °C.

### Establishment of a colitis model and treatment

**Figure 1.**
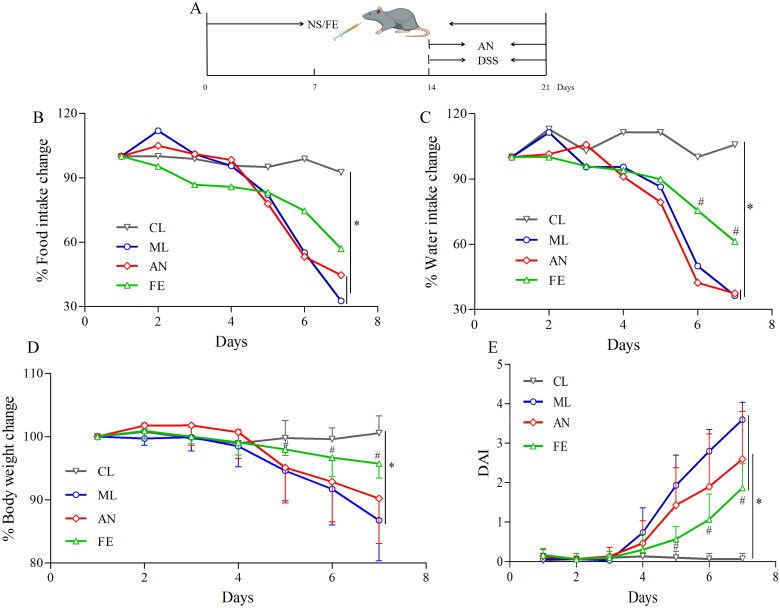
FLA decreased DSS-induced colitis in C57BL/6 mice. (A) Experimental design for evaluating the effects of FLA on DSS-induced colitis in mice. (B) Food intake change in each group. (C) Water intake change in each group. (D) Body weight change in each group. (E) Disease activity index (DAI). Data were indicated as the means ± SD (*n* = 5) and were analyzed using Student’s *t*-test; *P < 0.05 vs mice in CL group; #*P* < 0.01 vs mice in ML and AN group.

A model of acute colitis was created in mice by intragastric administration of 0.2 mL of DSS (0.6 g/mL) for 7 days. Mice were divided randomly into four groups. Mice in the control (CL) group were treated with physiologic (0.9%) saline for 21 days. Mice in the DSS (ML) group were treated with 0.9% saline for 14 days and then with DSS for 7 days. Mice in the positive control (AN) group were treated with 0.9% saline for 14 days and then with DSS and aspirin (0.1 g/kg) for 7 days. Mice in the SAF + DSS (FE) group were treated with SAF (0.85 g/kg) for 21 days and then with SAF and DSS for 7 days. Figure 1A indicates the grouping and the experimental time lines for all mice.

### Clinical scoring and histology

The weight of food consumed, the volume of water drunk and the bodyweight of mice were measured daily. The level of water and blood content in faeces was observed to monitor diarrhoea and rectal bleeding. Disease activity index (DAI) was calculated according to previous reports [23]. The mice were killed at the end of the experiment. The colon was acquired from the caecum to 1-cm above the anus. Then, the colon length under a constant load was measured. 4% paraformaldehyde in phosphate-buffered saline was used to fix colon tissues. The paraffin blocks were used to embed specimens to prepare haematoxylin and eosin (H&E)-stained sections of colon tissues, which were visualized under an optical microscope.

### Immune Organ Index

Mice in each group were weighed at the end of the experiment. The thymus gland and spleen were dissected, placed on filter paper to dry thoroughly and weighed. The Immune Organ Index was calculated using the following formula:

 $$\begin{array}{l} \text{Immune}\;\text{Organ}\;\;\text{Index}\;\;\left(\text{mg}/\text{g}\right)\;\\ =\;\text{Weight}\;\;\text{of}\;\;\text{immune}\;\;\text{organ}\;\;\left(\text{mg}\right)/\text{bodyweight}\;\;\left(\text{g}\right) \end{array}$$

### Haematology

A blood sample was collected from the retro-orbital sinus at the end of the experiment. After anticoagulation treatment, routine blood testing was carried out using a fully automatic blood analyzer (BC-5500; Mindray Medical, Shenzhen, China). Neutrophil count, % neutrophils, % lymphocytes, lymphocyte count, % lymphocytes, red blood cell (RBC) count, haematocrit, mean corpuscular volume (MCV), mean corpuscular haemoglobin (MCH), haemoglobin level, platelet count, mean platelet volume (MPV) and platelet distribution width (PDW) were documented.

### GM analysis

Caecal contents were collected at the end of the experiment. Total genomic DNA from all samples was extracted using the method based on cetyl trimethyl ammonium bromide. 16S rRNA genes of distinct regions were amplified using specific primers. Then, the products were purified and quantified. Sequencing libraries were generated using the TruSeq^®^DNA PCR-Free Sample Preparation Kit (Illumina, San Diego, CA, USA) following manufacturer recommendations, and index codes were added. Sequencing was done by Uparse software. Sequences with ≥97% similarity were assigned to the same operational taxonomic unit (OTU). A representative sequence for each OTU was screened for further annotation. Subsequent analysis of alpha diversity and beta diversity was undertaken based on these output-normalized data.

### Statistical analyses

Results are the mean ± standard deviation. The significance of the difference in data from the result of the control for each experimental test condition was calculated using the Student’s *t*-test. *P* < 0.05 was considered significant. QIIME software 1.9.1 was used to analyse alpha diversity and beta diversity.

## Results

### SAF attenuates DSS-induced colitis in C57BL/6 mice

To estimate the protective effect of SAF upon UC, a model of DSS-induced C57BL/6 colitis was established in mice by intragastric administration with 0.2 mL of DSS (6 g/kg bodyweight) for 7 days. The characteristics of DSS-induced colitis in C57BL/6 mice are bodyweight loss, diarrhoea and bloody stools, so these parameters were monitored daily. Compared with the CL group, the bodyweight was decreased in FE and especially in ML and AN groups (Figure 1D). SAF attenuated bodyweight loss significantly in comparison with that in ML and AN groups (Figure 1D). To investigate if the bodyweight loss was related to intake of food or water, we measured the mean water intake and food intake. In comparison with the CL group, the mean water intake and food intake were reduced slightly in the FE group (Figure 1B,C), whereas the mean water intake and food intake were reduced significantly in ML and AN groups (Figure 1B,C). These findings suggested that SAF could improve bodyweight loss of UC mice induced by DSS by reducing the suppression of appetite.

**Figure 2.**
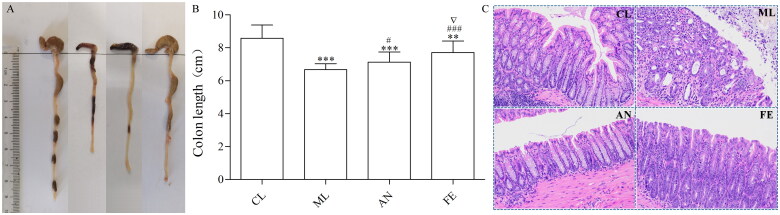
(A,B) The lengths of colons of mice in each group. (C) The colons from each experimental group were processed for histological evaluation (H&E staining, 200×). Data were indicated as the means ± SD and were analysed using Student’s *t*-test; **P < 0. 01 and ***P < 0.001 vs mice in CL group, ^#^*P* < 0.05 and ^###^*P* < 0.001 vs mice in ML group, ^∇^*P* < 0.05 vs mice in AN group.

The DAI reflects the severity of bodyweight loss, blood in stools and stool consistency. Comparing with the control group, the DAI had an obvious increase in the ML, AN and FE groups. The DAI score was obviously higher in the ML and AN groups than that in the FE group (Figure 1E). The length of the colon was shortened markedly in ML groups, however, SAF improved this change (Figure 2A,B). H&E staining further estimated the severity of colonic inflammation (Figure 2C). The intact surface epithelium, crypt glands and submucosa could be seen in the CL group (Figure 2C). In the AN group and especially the ML group, damage to the surface epithelium, disruption of crypt glands and infiltration of inflammatory cells were observed. The FE group showed more intact surface epithelium and crypt glands than that in the ML or AN groups, which suggested that SAF alleviated colitis induced by DSS in C57BL/6 mice.

### SAF modifies blood parameters in mice with DSS-induced colitis

Changes of blood parameters were detected in different groups (Table 1). In comparison with CL group, significant differences of almost all blood parameters were observed in the ML group, whereas only some parameters showed significant differences in the AN group (neutrophil count, % neutrophils, % lymphocytes, RBC count, haematocrit, haemoglobin level, MPV) and FE group (% neutrophils, % lymphocytes, RBCs, haemoglobin). However, compared with the ML group, there were no significant differences in blood parameters in the AN group. Most parameters (% neutrophils, % lymphocytes, RBC count, haematocrit, MCV, MCH, haemoglobin level, platelet count, MPV, PDW) displayed obvious differences in the FE group. MCV, MCH and MPV demonstrated significant differences between the AN group and FE group. These results suggested that SAF improved blood parameters in mice with DSS-induced UC.

**Table 1.** The levels of blood routine results in all groups.

Detection index	CL	ML	AN	FE
WBC (×10^9^/L)	7.56 ± 1.15	23.31 ± 13.91*	10.62 ± 7.62	8.41 ± 1.06
Neu (×10^9^/L)	1.06 ± 0.168	8.53 ± 5.06*	1.99 ± 0.64*	1.85 ± 0.88^#^
Neu% (%)	13.97 ± 0.58	35.07 ± 8.74**	27.47 ± 7.81**	20.77 ± 3.41^*,#^
Lym (×10^9^/L)	6.50 ± 0.99	15.20 ± 8.96*	6.97 ± 3.33	6.11 ± 0.86
Lym% (%)	85.90 ± 0.56	64.57 ± 8.67**	75.08 ± 12.33*	77.72 ± 4.98^*,#^
RBC (×10^12^/L)	10.06 ± 0.56	3.06 ± 0.82***	7.42 ± 1.92*	9.07 ± 0.72^*,###^
HCT (%)	42.25 ± 2.08	14.73 ± 3.46***	33.00 ± 7.53*	38.90 ± 2.83^###^
MCV (fL)	42.03 ± 0.57	47.48 ± 2.85**	44.82 ± 2.13	40.95 ± 2.04^#,Δ^
RDW-CV	15.55 ± 0.69	18.00 ± 1.09*	17.47 ± 2.00	16.42 ± 1.52
RDW-SD (fL)	28.28 ± 1.49	35.77 ± 3.59**	32.95 ± 4.52	31.37 ± 2.03^#^
HGB (g/L)	142.17 ± 8.47	63.17 ± 27.53***	106.83 ± 27.15*	128.17 ± 10.46^*,###^
MCH (pg)	14.13 ± 0.17	15.05 ± 0.71*	14.45 ± 0.28	14.12 ± 0.12^#,Δ^
MCHC (g/L)	336.00 ± 5.57	318.00 ± 20.38	324.83 ± 5.87	328.83 ± 4.88
PLT (×10^9^/L)	1287.83 ± 147.31	559.00 ± 120.34***	1137.67 ± 247.71	1149.17 ± 206.15^###^
MPV (fL)	5.07 ± 0.13	6.83 ± 0.65**	5.78 ± 0.24**	5.25 ± 0.08^##,ΔΔ^
PDW	14.92 ± 0.07	14.68 ± 0.09**	14.83 ± 0.13	14.88 ± 0.07^#^
PCT (%)	0.65 ± 0.08	0.38 ± 0.06***	0.64 ± 0.09	0.54 ± 0.13

Data were indicated as the means ± SD and were analysed using Student’s *t*-test; *P < 0.05, **P < 0.01 and ***P < 0.001 vs mice in CL group; ^#^*P* < 0.05, ^##^*P* < 0.01 and ^###^*P* < 0.001 vs mice in ML group; ^Δ^*P* < 0.05, ^ΔΔ^*P* < 0.05 vs mice in AN group.

### GM composition

**Figure 3.**
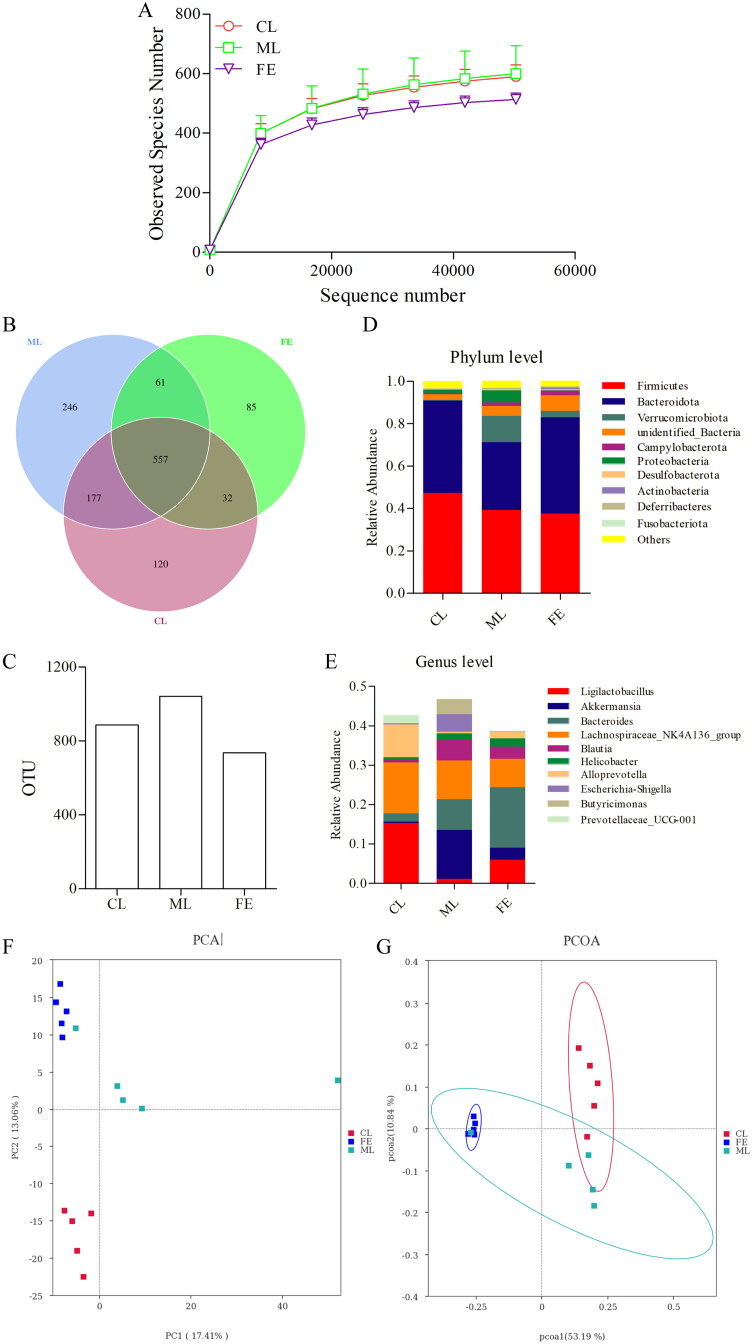
Effects of FLA on gut microbial dysbiosis. (A) Rarefaction curve of GM from CL, ML, AN and FE group. (B,C) The Venn diagrams indicate the numbers of OTUs in CL, ML and FE group. (D,E) Relative abundance of GM from the caecal contents of all the groups at the phylum and genus levels, classified by the representation of top 10 species with maximum abundance. (F,G) Beta diversity changes calculated by PCoA based on the OTU abundance.

We carried out sequencing of bacterial 16S rRNA from caecal samples using the NovaSeq™ platform (Illumina) to estimate the regulatory effect of SAF on the GM. Rarefaction curves describe sample diversity within groups. They can reflect the rationality of sequencing data directly and reflect the abundance of species in a sample indirectly. The volume of sequencing data appeared to be sufficient according to Figure 3A.

A total of 886 OTUs were found in the CL group, 1041 OTUs in the ML group and 735 OTUs in the FE group (Figure 3C). Venn diagrams showed that 557 OTUs were shared by all groups, whereas 734 OTUs were shared between the CL group and ML group, 589 OTUs between the CL group and FE group and 618 OTUs between the ML group and FE group (Figure 3B). There were 120 unique OTUs in the CL group, 246 unique OTUs in the ML group and 85 unique OTUs in the FE group (Figure 3B).

The top-10 species with maximum abundance at phylum and genus levels were analysed to estimate the overall structure of the bacterial community in different groups. Bacteria from the phyla Firmicutes and Bacteroidetes were the most abundant in all groups. The relative abundance of bacteria from the phyla Verrucomicrobiota and Proteobacteria was increased markedly in the ML group compared with that in the CL group. After SAF had been administered, these changes were reversed (Figure 3D).

Bacteria from 10 major genera were observed in all groups (Figure 3E). The abundance of *Ligilactobacillus* decreased, whereas that of *Akkermansia*, *Blautia*, *Escherichia-Shigella* and *Butyricimonas* increased in the ML group compared with the CL group. After SAF treatment, these changes were reversed (Figure 3E). Overall, these results showed that SAF corrected the GM dysbiosis induced by DSS.

PCA is an applied variance decomposition based on Eucandean distance. principal component analysis (PCA) indicated distinct clustering of GM composition for the CL group, ML group and FE group (Figure 3F). Based on the unweighted UniFrac distance, principal coordinate analysis (PCoA) also displayed distinct clustering of GM composition for the CL group, ML group and FE group (Figure 3G). The findings of PCA and PCoA suggested that SAF induced obvious changes in GM composition.

### Statistical variation among groups

Analysis of similarities (ANOSIM) and permutational multivariate analysis of variance [MANOVA (ADONIS)] were used to investigate statistical differences among groups. ANOSIM is employed to ascertain if a difference between groups is significantly greater than that within groups [24]. ANOSIM was applied to detect if grouping was meaningful. ANOSIM suggested that the interspecific variation among the three groups was greater than the intraspecific variation (*R* > 0), and that the variation in GM composition among the four groups was significant (*P* < 0.05; Table 2). ADONIS is a non-parametric MANOVA method based on the Bray–Curtis distance [25]. ADONIS can be used to analyse the interpretation of sample-based differences by different grouping factors, and analyse the statistical significance of a grouping using the permutation test. ADONIS results were similar to ANOSIM data (Table 3).

**Table 2.** Analysis of ANOSIM and ADONIS of gut microbiota among the CL, ML and FE group.

	ANOSIM		ADONIS	
	*R*	*P*	*R* ^2^	*P*
CL–ML	1	0.007	0.540 (0.460)	0.001
CL–FE	0.992	0.013	0.481 (0.519)	0.001
ML–FE	0.772	0.009	0.386 (0.614)	0.001

**Table 3.** Differential species between CL groups and ML groups in phylum, class, order, family and genus levels.

Phylum	Class	Order	Family	Genus
Verrucomicrobiota	Verrucomicrobiae	Verrucomicrobiales	Akkermansiaceae	*Akkermansia*
Firmicutes	Bacilli	Lactobacillales	Lactobacillaceae	*Ligilactobacillus*
Firmicutes	unidentified_Firmicutes	Oscillospirales	Ruminococcaceae	–
Firmicutes	Clostridia	Peptostreptococcales-Tissierellales	Peptostreptococcaceae	*Romboutsia*
Firmicutes	Clostridia	Clostridia_vadinBB60_group	unidentified_Clostridia_vadinBB60_group	unidentified_Clostridia_vadinBB60_group
Firmicutes	Clostridia	Lachnospirales	Lachnospiraceae	[Eubacterium]_xylanophilum_group
Firmicutes	Clostridia	Lachnospirales	Lachnospiraceae	[Eubacterium]_fissicatena_group
Firmicutes	Clostridia	Oscillospirales	Oscillospiraceae	NK4A214_group
Bacteroidota	Bacteroidia	Bacteroidales	Muribaculaceae	*Muribaculum*
Bacteroidota	Bacteroidia	Bacteroidales	Prevotellaceae	Prevotellaceae_UCG-001
Bacteroidota	Bacteroidia	Bacteroidales	Prevotellaceae	*Alloprevotella*
Bacteroidota	Bacteroidia	Bacteroidales	Rikenellaceae	*Rikenella*
Bacteroidota	Bacteroidia	Bacteroidales	Rikenellaceae	Rikenellaceae_RC9_gut_group
Bacteroidota	Bacteroidia	Bacteroidales	Marinifilaceae	*Butyricimonas*
unidentified_Bacteria	Alphaproteobacteria	Rhodospirillales	–	–

The colour-deepened parts represent differential species present at the corresponding level.

**Figure 4.**
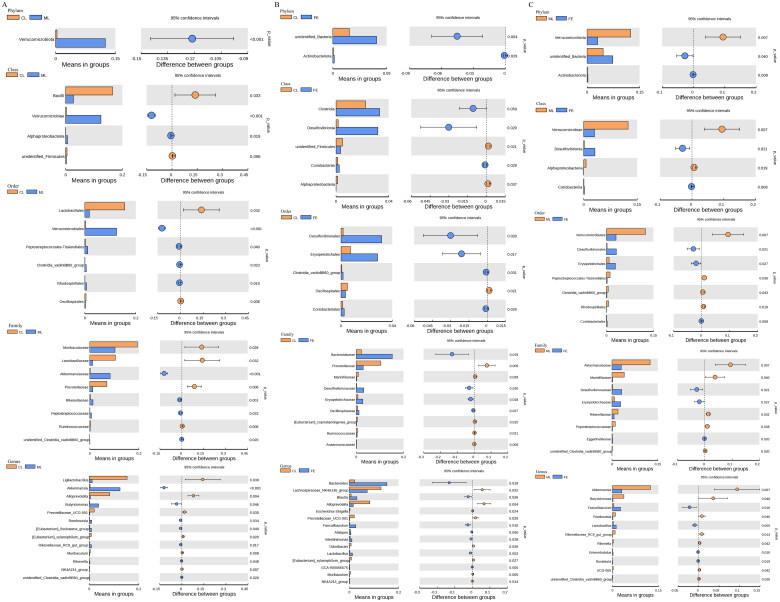
The diagram indicates the categories of species that are obviously different between the two groups, (A) CL group vs ML group, (B) CL group vs FE group and (C) ML group vs FE group.

To search for different species, Student’s *t*-test tests were carried out under classification levels (phylum, class, order, family, genus) and were found to be different between groups (Figure 4 and Tables 3–5). One phylum was distinctly different between CL and ML groups. Two phyla were obviously different between CL and FE groups. Four phyla were significantly different between ML and FE groups. The abundance of bacteria of the phylum Verrucomicrobiota was not significantly different between CL and FE groups, but the abundance of Verrucomicrobiota was obviously increased in UC mice induced by DSS, whereas SAF treatment reversed this change.

**Table 4.** Differential species between CL groups and FE groups in phylum, class, order, family and genus levels.

Phylum	Class	Order	Family	Genus
Actinobacteriota	Coriobacteriia	Coriobacteriales	–	–
unidentified_Bacteria	Desulfovibrionia	Desulfovibrionales	Desulfovibrionaceae	–
Firmicutes	unidentified_Firmicutes	Oscillospirales	Ruminococcaceae	–
Firmicutes	Clostridia	Oscillospirales	Oscillospiraceae	*Intestinimonas*
Firmicutes	Clostridia	Oscillospirales	Oscillospiraceae	NK4A214_group
Firmicutes	Clostridia	Oscillospirales	Oscillospiraceae	–
Firmicutes	Clostridia	Clostridia_vadinBB60_group	–	–
Firmicutes	Clostridia	Oscillospirales	[Eubacterium]_coprostanoligenes_group	–
Firmicutes	Clostridia	Peptostreptococcales-Tissierellale	Anaerovoracaceae	–
Firmicutes	Clostridia	Lachnospirale	Lachnospiraceae	Lachnospiraceae_NK4A136_group
Firmicutes	Clostridia	Lachnospirale	Lachnospiraceae	[Eubacterium]_xylanophilum_group
Firmicutes	Clostridia	Lachnospirale	Lachnospiraceae	GCA-900066575
Firmicutes	Clostridia	Lachnospirale	Lachnospiraceae	*Blautia*
Firmicutes	Bacilli	Erysipelotrichales	Erysipelotrichaceae	*Faecalibaculum*
Firmicutes	Bacilli	Lactobacillales	Lactobacillaceae	*Lactobacillus*
Proteobacteria	Alphaproteobacteria	–	–	–
Proteobacteria	Gammaproteobacteria	Enterobacterales	Enterobacteriaceae	*Escherichia-Shigella*
Bacteroidota	Bacteroidia	Bacteroidales	Bacteroidaceae	*Bacteroides*
Bacteroidota	Bacteroidia	Bacteroidales	Prevotellaceae	Prevotellaceae_UCG-001
Bacteroidota	Bacteroidia	Bacteroidales	Prevotellaceae	*Alloprevotella*
Bacteroidota	Bacteroidia	Bacteroidales	Marinifilaceae	*Odoribacter*
Bacteroidota	Bacteroidia	Bacteroidales	Rikenellaceae	*Alistipes*
Bacteroidota	Bacteroidia	Bacteroidales	Muribaculaceae	*Muribaculum*

The colour-deepened parts represent differential species present at the corresponding level.

**Table 5.** Differential species between ML groups and FE groups in phylum, class, order, family and genus levels.

Phylum	Class	Order	Family	Genus
Verrucomicrobiota	Verrucomicrobiae	Verrucomicrobiales	Akkermansiaceae	*Akkermansia*
unidentified_Bacteria	Desulfovibrionia	Desulfovibrionales	Desulfovibrionaceae	–
unidentified_Bacteria	Alphaproteobacteria	Rhodospirillales	–	–
Actinobacteriota	Coriobacteriia	Coriobacteriales	Eggerthellaceae	*Enterorhabdus*
Firmicutes	Bacilli	Erysipelotrichales	Erysipelotrichaceae	*Faecalibaculum*
Firmicutes	Bacilli	Lactobacillales	Lactobacillaceae	*Lactobacillus*
Firmicutes	Clostridia	Peptostreptococcales-Tissierellales	Peptostreptococcaceae	*Romboutsia*
Firmicutes	Clostridia	Clostridia_vadinBB60_group	unidentified_Clostridia_vadinBB60_group	unidentified_Clostridia_vadinBB60_group
Firmicutes	Clostridia	Oscillospirales	Oscillospiraceae	UCG-005
Firmicutes	Clostridia	Lachnospirales	Lachnospiraceae	*Roseburia*
Bacteroidota	Bacteroidia	Bacteroidales	Rikenellaceae	Rikenellaceae_RC9_gut_group
Bacteroidota	Bacteroidia	Bacteroidales	Rikenellaceae	*Rikenella*
Bacteroidota	Bacteroidia	Bacteroidales	Marinifilaceae	*Butyricimonas*

The colour-deepened parts represent differential species present at the corresponding level.

Similar phenomena were displayed in terms of class (Verrucomicrobiota), order (Verrucomicrobiales, Pepto­streptococcales-Tissierellales, Rhodospirillales), family (Akkermansiaceae, Rikenellaceae, Peptostrept­ococcaceae, unidentified_Clostridia_vadinBB60_group) and genus (*Akkermansia*, *Butyricimonas*, *Romboutsia*, Rikenellaceae_RC9_gut_group, *Rikenella*, unidentified_Clostridia_vadinBB60_group) levels. In conclusion, it appeared to be very important to ameliorate colitis for bacteria of the phylum Verrucomicrobiota, class Verrucomicrobiae, order Verrucomicrobiales, family Akkermansiaceae and genus *Akkermansia*.

**Figure 5.**
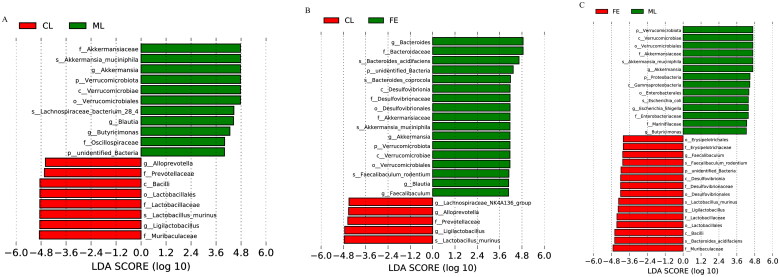
The LEfSe analysis of the GM differed between two groups, (A) CL group vs ML group, (B) CL group vs FE group and (C) ML group vs FE group. The statistical test was performed using LDA effect size method.

To identify GM composition in detail, linear discriminant analysis effect size (LEfSe) was carried out. LEfSe is a tool for discovering and interpreting high-dimensional biological identifiers (genes, pathways, taxa) that can be used to compare ≥2 groups, emphasize statistical significance and biological relevance and enable the discovery of biomarkers with singificant differences between groups. In comparison with CL group, the GM, as well as bacteria of the phylum Verrucomicrobiota, class Verrucomicrobiae, order Verrucomicrobiales, family Akkermansiaceae and genus *Akkermansia* were significant biomarkers in mice with DSS-induced UC (Figure 5). However, the biomarkers of GM changed after SAF treatment (Figure 5). This result was in accordance with the results of Student’s *t*-tests. Taken together, DSS-induced UC resulted in GM dysbiosis in mice, but SAF could regulate GM dysbiosis to reconstruct intestinal homeostasis.

## Discussion

Most medications used to treat colitis are efficacious only temporarily [24]. Natural extract from medicinal plants/herbs have become an important complementary treatment for UC [26]. Previously, we demonstrated that *S. arvensis L.* extract had a protective effect against DSS-induced UC in mice [27]. Flavones are major constituents of *S. arvensis* L. extract. Here, we investigated if SAF could ameliorate colitis, and we postulated the underlying molecular mechanism of action. DSS was applied to construct a colitis model in mice because a colitis model induced by DSS mice is similar to UC experienced in humans [28]. The important parameters of estimating the level of inflammation in UC are bodyweight loss, DAI score, colon length and pathologic changes [29]. SAF had a protective effect against DSS-induced UC in mice (Figures 1 and 2).

Studies have shown that patients with UC have abnormal numbers of inflammatory cells and altered blood coagulation [30]. Often, neutrophil infiltration is seen in tissue biopsies of UC patients. Neutrophils can release adhesion molecules, proteases and interleukins. These proinflammatory mediators regulate the inflammatory process alone or in concert [31]. Neutrophils are involved in the development of tissue injury and inflammation, and secrete cytokines that lead to additional tissue damage [32]. The neutrophil count was increased significantly in ML and AN groups in comparison with that in the CL group (Table 1). Lymphocytes are involved in the immune response through cell/humoral regulation, and the lymphocyte count is altered in UC patients [33]. The lymphocyte count was increased in the ML group compared with that in the CL group (Table 1). Platelets can promote haemostatic reactions, repair endothelial tissue and participate in inflammatory reactions in the body. In recent years, changes in the platelet count in UC patients have attracted considerable interest. Several studies have demonstrated that the platelet count is increased in UC patients [34], and our results were similar (Table 1). Anaemia is a common complication of IBD and may be associated with chronic blood loss and nutrient consumption in UC. The RBC count and haemoglobin level were reduced significantly in ML, AN and FE groups compared with those in the CL group, whereas these two parameters increased in FE group in comparison with ML group (Table 1). These results suggested that SAF improved blood parameters in mice with DSS-induced UC.

The GM is a micro-ecosystem which has a vital role in maintaining human health [4]. Scholars have suggested that GM composition is changed in IBD patients in comparison with that in healthy humans [35,36]. This phenomenon is manifested as an imbalance in the ratio of pathogenic bacteria: normal bacteria, and an increased abundance of pathogenic bacteria. Therefore, the GM is a possible target to treat IBD. Previously, we suggested that the protective effects of *S. arvensis* L. extract on the mice of colitis induced by DSS may have a connection with GM diversity, so we investigated if SAF improved colitis in mice by adjustment of GM diversity. Amplicon sequencing of 16S rRNA has become an important method for studying the composition and structure of microbial communities in samples. ANOSIM and ADONIS suggested that variations in GM composition among the four groups were significant (Table 2). Interestingly, we found that it was very important to ameliorate colitis for bacteria of the phylum Verrucomicrobiota, class Verrucomicrobiae, order Verrucomicrobiales, family Akkermansiaceae and genus *Akkermansia*. The results of LEfSe were similar to the data of Student’s *t*-tests (Figure 5). Taken together, the present study suggested that DSS-induced UC resulted in GM dysbiosis in mice, and that SAF could regulate GM dysbiosis to reconstruct intestinal homeostasis.

## Conclusions

SAF could improve DSS-induced bodyweight loss and the DAI score and ameliorate pathologic damage, which suggested that SAF had a protective effect against DSS-induced UC in mice. SAF improved the blood parameters of DSS-induced UC in mice. 16S rRNA sequencing suggested that it was very important to ameliorate colitis for bacteria of bacteria of the phylum Verrucomicrobiota, class Verrucomicrobiae, order Verrucomicrobiales, family Akkermansiaceae and genus *Akkermansia*. The protective effects of SAF on the mice of colitis induced by DSS may have a connection with GM diversity.

## Data Availability

Data that support this article are available within the article.
